# Changes in lifestyles, cognitive impairment, quality of life and activity day living after combined use of smartphone and smartband technology: a randomized clinical trial (EVIDENT-Age study)

**DOI:** 10.1186/s12877-022-03487-5

**Published:** 2022-10-06

**Authors:** José I. Recio-Rodríguez, Susana Gonzalez-Sanchez, Olaya Tamayo-Morales, Manuel A. Gómez-Marcos, Luis Garcia-Ortiz, Virtudes Niño-Martín, Cristina Lugones-Sanchez, Emiliano Rodriguez-Sanchez

**Affiliations:** 1 Unidad de Investigación de Atención Primaria de Salamanca (APISAL), Instituto de Investigación Biomédica de Salamanca (IBSAL), Red de investigación en cronicidad, Atención Primaria Y Promoción de La Salud (RICAPPS) (RD21/0016), Gerencia de Atención Primaria de Salamanca, Gerencia Regional de Salud de Castilla Y León (SACyL), Salamanca, Spain; 2 Facultad de Enfermería Y Fisioterapia, Universidad de Salamanca, Donantes de Sangre S/N, 37007 Salamanca, Spain; 3 Departamento de Medicina, Universidad de Salamanca, Salamanca, Spain; 4 Departamento de Ciencias Biomédicas Y del Diagnóstico, Universidad de Salamanca, Salamanca, Spain; 5 Facultad de Enfermeria, Universidad de Valladolid, Valladolid, Spain

**Keywords:** Older adult, Physical activity, Nutrition, Body composition, Quality of life

## Abstract

**Background:**

The aim of this study was to assess the efficacy of the combined use of smartphone and smartband technology for 3-months alongside brief lifestyle counselling, versus counselling alone, in increasing physical activity. As secondary objectives, the effects of the intervention on dietary habits, body composition, quality of life, level of functionality and cognitive performance were assessed.

**Methods:**

This study employed a randomized clinical trial of two-parallel groups design – control group (CG) and intervention group (IG). The study was conducted in 3 Spanish health-centres between October 2018-February 2020. Eligible participants were people of both sexes and aged between 65–80 years attending the health-centres with a score ≥ 24 points on the Mini-Mental State Examination. Key variables included physical activity, dietary pattern, body composition, cognitive performance, level of functionality and quality of life. All variables were measured at baseline and after 3-months. Both groups received a brief nutritional and physical activity advice. Intervention group participants were instructed to use a smartphone application for a period of 3-months. This application integrates information on physical activity received from a fitness bracelet and self-reported information on the patient’s daily nutritional composition.

**Results:**

The study population comprised 160 participants (IG = 81, CG = 79), with a mean age of 70.8 ± 4.0 years (61.3% women). No difference was found in the primary and secondary outcomes analyzed (physical activity (steps/min -0.4 (-1.0 to 0.2) *p* = 0.174), and dietary habits (Mediterranean diet score 0.0 (-0.6 to 0.6) *p* = 0.956) that could be attributed to either group after an ANCOVA test. A difference attributable to the intervention was observed in the total Clock test score (0.7 (0.1 to 1.2) *p* = 0.018.

**Conclusions:**

In a sample of people over 65 years of age, the combined use of the EVIDENT 3 smartphone app and an activity tracking bracelet for 3-months did not result in lifestyles changes related to the amount and level of physical activity or the eating habits, compared to brief lifestyle advice. Other clinical parameters were not changed either, although at the cognitive level, a slight improvement was observed in the score on the Clock test assessing a variety of cognitive functions such as memory.

**Trial registration:**

The study was registered in ClinicalTrials.gov Identifier: NCT03574480. Date of trial Registration 02/07/2018.

## Background

The World Health Organization defines active aging as making the most of opportunities to achieve physical, mental and social well-being throughout life with the aim of extending quality of life, productivity and life expectancy at advanced ages and with a minimum prevalence of disability [1]. One of the tools to achieve this goal is lifestyle improvement, including habits related to physical activity or diet. Thus, for example, increased physical activity in older people assessed through objective methods has been associated with a lower incidence of cardiovascular events [2]. Adherence to the Mediterranean Diet (MD) has been linked to better cognitive performance, in addition to other beneficial effects such as the reduction of signs of frailty or depression in older people [3]. Meeting these objectives could be achieved through prevention and health promotion interventions or programs that include innovative and efficient solutions, such as the use of various digital technologies applied to health [4].

The effectiveness of interventions with e-Health (set of Information and Communication Technologies (ICTs) that, as tools, are used in the health environment in terms of prevention, diagnosis, treatment, follow-up) or m-Health (mobile health) components on aspects related to healthy aging has been addressed by Buyl R. et al. [5] in a systematic review which found that both the study population, the type of intervention and the results obtained were very heterogeneous in the 14 studies included. Most studies obtained positive results, although of low intensity, highlighting benefits for the level of physical activity, some clinical parameters such as blood pressure and positive results in aspects related to the assessment of cognitive performance, such as memory. The effect of this type of multimodal intervention has also been analyzed in relation to better daily functioning and greater functional independence [6]. Increasing competence in using these new technologies has been linked to better quality of life in the elderly [7].

e-Health and m-Health have been used both within health promotion programs for older adults and outside these formal programs [8]. Some studies have analyzed the barriers and facilitators older people have in the use of these digital technologies [6, 9] and show that both the motivation of the users and the population at which the tool is aimed, as well as the level of support in its use and the self-efficacy strategies employed, seem to be determining factors [8].

The aim of this study was to assess the efficacy of the combined use of smartphone and smartband technology for three months alongside brief lifestyle counselling, versus counselling alone, in increasing physical activity. As secondary objectives, the effects of the intervention on dietary habits, body composition, quality of life, activities of daily living and cognitive performance were assessed.

## Methods

### Trial design and setting

This study was a controlled and randomized clinical trial of two parallel groups carried out in three health centres in the cities of Salamanca and Valladolid in Spain. The research was carried out between October 2018 and February 2020. The study was registered (Date of trial Registration 02/07/2018). in ClinicalTrials.gov Identifier: NCT03574480 and the research protocol was published [10].

### Sample size estimation

Sample size was estimated for the main study variable (Physical activity-number of steps). The calculation of sample size was based on the work of Alonso-Dominguez et al. [11], who used the same smartphone application in his study and whose design is similar to the one presented here. Assuming a standard deviation of 3847 steps/day (2.6 steps/min), a power of 0.8 and an alpha risk of 0.05 in a bilateral test, 80 participants are necessary in each group to detect an increase of 1850 steps/day in the experimental group compared to the control group estimating a 15% dropout rate.

### Participants and randomization

**Fig. 1:**
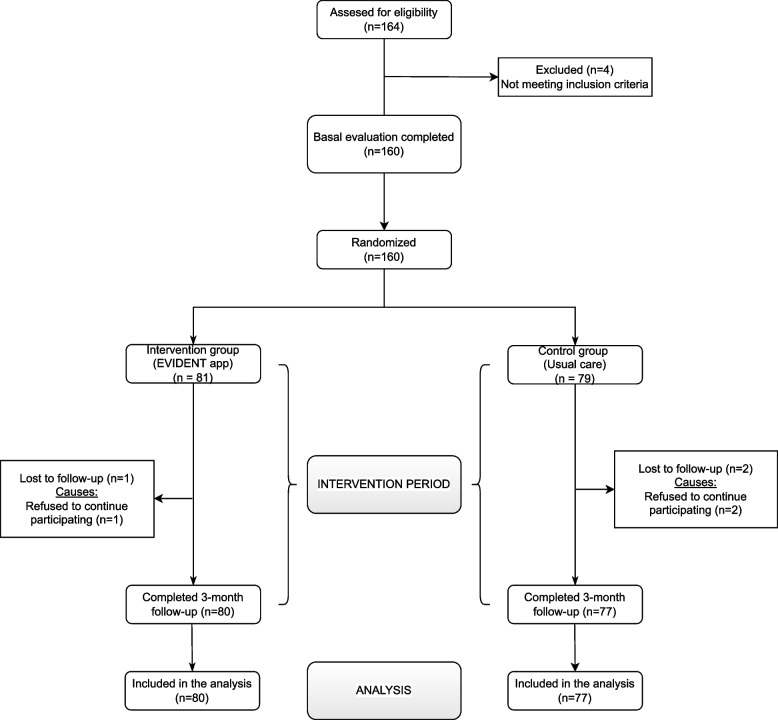
Study flowchart

A total of 164 participants were selected in the primary care services of the participating health centres through consecutive sampling. Two inclusion criteria were applied: 1) people of both sexes and aged between 65 and 80 years attending the health centres; 2) a score greater than or equal to 24 points on the Mini-Mental State Examination (MMSE). Exclusion criteria were: 1) not being able to perform physical activity or follow a MD; 2) presence of coronary or cerebrovascular atherosclerotic disease, grade II or higher heart failure according to the New York Heart Association (NYHA) criteria, moderate or severe chronic obstructive pulmonary disease, musculoskeletal disease that limits walking, kidney disease or advanced liver disease, severe mental illness and oncological disease under treatment and diagnosed in the last 5 years, and being in a terminal situation. After applying the selection criteria, four participants were excluded and 160 signed the informed consent and performed the baseline evaluation. After this baseline evaluation, participants were randomly assigned to the intervention group (IG) (*n* = 81) or the control group (CG) (*n* = 79). The allocation sequence was generated by an independent investigator using the Epidat 4 program [12] (Fig. 1).

### Intervention

Common intervention in both groups: Prior to the random group assignment, all participants (control and intervention) received nutritional advice aimed at good adherence to the MD. The counselling consisted of a 10-min individual appointment in which the concepts of the diet were explained and aspects such as health benefits and evidence generated by this nutritional pattern were discussed. Both groups also received brief advice on physical activity aimed at complying with the current recommendations for the general population and the elderly population, i.e., performing at least 30 min of moderate activity, five days a week, or 20 min of vigorous activity, 3 days a week. At the end of this counselling session, participants were given an information leaflet.

**Fig. 2:**
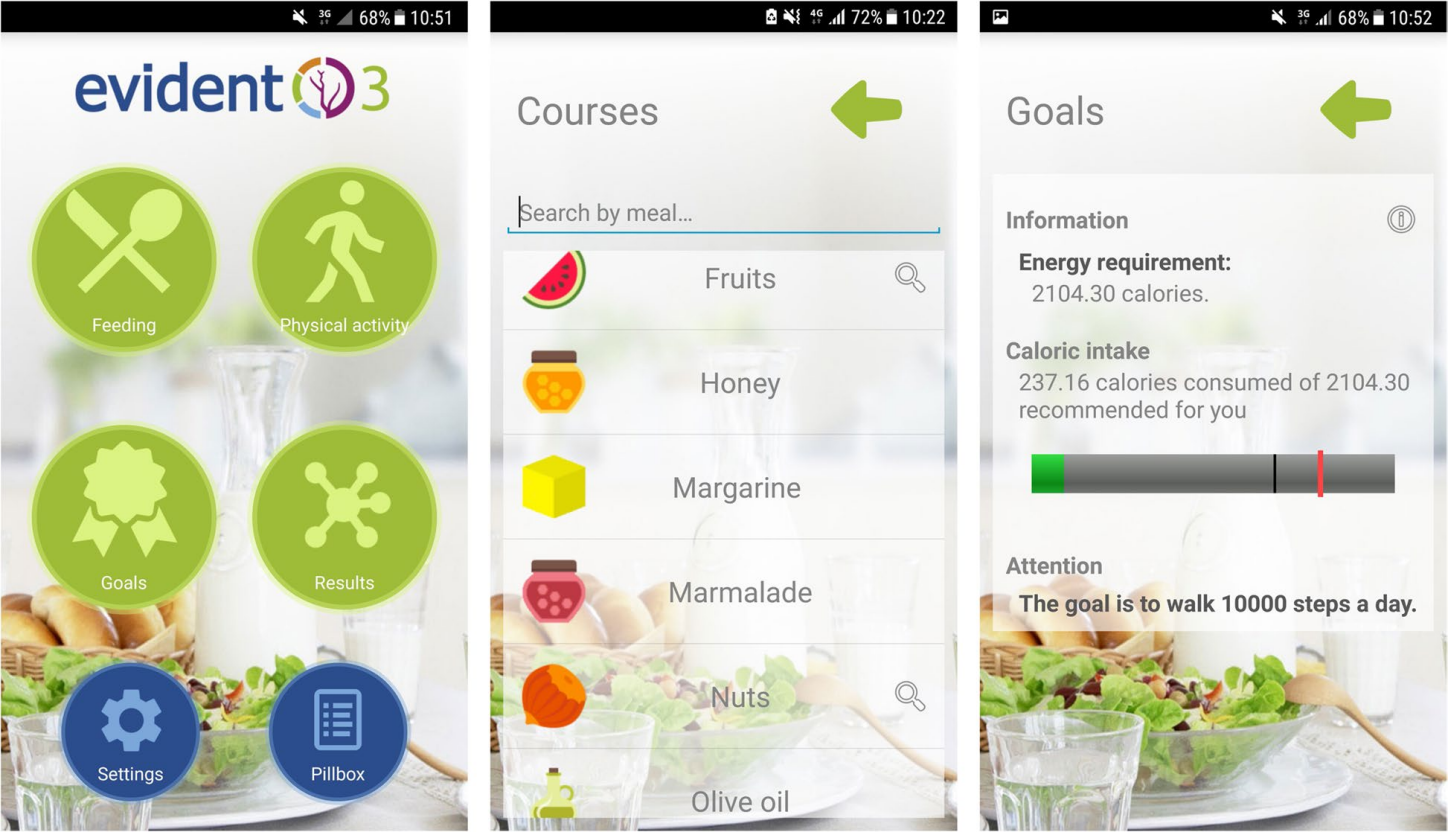
Screenshots of the EVIDENT 3 smartphone application

Specific intervention in the experimental group: Participants in the intervention group were given a smartphone (Samsung Galaxy J3) and a smartband (Xiaomi Miband S2) on loan with the EVIDENT 3 (Lifestyle and arterial stiffness study) app pre-installed and instructions for its use during a period of 3 months (90 days). Each participant had a visit of approximately 15 min in which they were individually trained in handling the device and the app developed in the EVIDENT 3 study [13] (Intellectual property registration No. 00/2017/2438) and installed on the smartphone. In the first part of this visit, the app was configured with personalized data regarding age, sex, weight and height, to allow the daily energy needs of each participant to be calculated. The app allows daily monitoring of food and physical activity by integrating the information collected by the smartband in the form of steps, calories and heart rate (physical activity) and food and dishes eaten as recorded daily by the participant in selecting from the application menu (Fig. 2). At the end of the day, the application makes recommendations and a personalized plan to improve eating habits and physical activity over the following days. At the three-month visit, the devices were returned, the information was downloaded, and the number of days on which the application was used was calculated, thus establishing the adherence percentage, with a value of 100% for a total of 90 days of use (the maximum).

### Variables and measurement instruments

#### Primary outcome

##### Physical activity

The main variable for measuring physical activity was the change in the amount and intensity of physical activity, expressed in number of steps measured by accelerometer. This device also detects the total daily time spent in light, moderate and intense physical activity and the kilocalories expended. Validated GT3X accelerometers were used [14]. The participants wore the accelerometer on the right side of the waist, attached by elastic belt, for seven consecutive days. The data was recorded minute by minute. If the accelerometer registered 10 consecutive zeros over 10 min, the measurement was considered null. The intensity of physical activity (low, moderate or high) was determined according to the cut-off points proposed by Freedson [15]. Physical activity was also measured on a self-reported basis with the International Physical Activity Questionnaire (IPAQ). The short version, validated in Spanish, was used to evaluate the physical activity of the last seven days [16]. This questionnaire classifies adult populations according to their level of activity (low, moderate and high) and differentiates between three types of activity: walking, moderate activity and vigorous activity. The total amount of physical activity performed was estimated in METs/minute/week.

#### Secondary outcomes

##### Sedentarism

The number of hours per day spent sitting was collected using the questionnaire developed by Marshall et al. [17], which evaluates the hours that the individual remains seated in their work activity, in commuting and in leisure activities related to the use of screens (television, computer, tablets/smartphone). The questionnaire differentiates between working and non-working days.

##### Dietary pattern

Change in adherence to the MD was measured through the total score (baseline and final) in the Mediterranean Diet Adherence Questionnaire. This questionnaire, validated in Spain, was used in the PREDIMED study [18] and has 14 questions regarding adherence to the MD. Each question was scored with zero or one point. One point was given for using olive oil as the main fat for cooking, preferring white meat to red meat, daily consumption of four or more tablespoons (one tablespoon = 13.5 g) of olive oil (including oil used for frying, dressing salads, etc.), two or more servings of vegetables, three or more pieces of fruit, less than one serving of red meat or sausage, less than one serving of animal fat, less than one cup (one cup = 100 ml) of carbonated and/or sugary drinks; and also for weekly intake of seven or more glasses of wine, three or more servings of legumes, three or more servings of fish, two items or fewer of shop-bought pastries, three or more servings of nuts, two or more helpings of sofrito, (traditional sauce made with tomato, garlic, onion or leeks, sautéed with olive oil). The final score ranges from zero to fourteen points.

##### Body composition

This was recorded using the Inbody 230 bioelectric impedance analyzer [19] which provides data on body weight, skeletal muscle mass, fat mass, total body water, fat-free mass, and percentage of body fat. Height was measured with a portable system (Seca 222), with the average of two measurements being recorded.

##### Cognitive performance

General cognitive status was assessed by applying the Spanish validation of the MMSE [20]. This test makes it possible to track performance in different cognitive functions: temporal-spatial orientation, attention, learning and memory, executive functions, language and visuoconstructive abilities. The score can range from 0 to 30 points, with normal cognitive performance considered to be a score over 24 points. In addition, the Clock test was applied [21] to assess visuoconstructive skills, executive functions and language. Finally, verbal fluency ability was measured by a one-minute categorical fluency test of animal naming [22].

##### Activities of daily living

The level of functionality was assessed with the Functional Activities Questionnaire (FAQ), which was designed by Pfeffer and is based on a previous study by the same author [23, 24]. This is a screening scale for mild cases of dementia through questions that assess the ability to carry out complex social activities, the so-called instrumental activities of daily living. Participants are asked about the following 11 aspects: managing money, shopping, making tea or coffee and turning off the stove, cooking, knowing the news in one’s community, understanding and discussing radio and television news, reading magazines and books, remembering appointments and important dates, managing medication, traveling alone outside one’s neighbourhood, greeting friends and going out alone safely. Each item scores from 0 (normal) to 3 (totally dependent).

##### Quality of life

Quality of life was assessed using a modified version of the World Health Organization quality of life (WHOQOL) instrument called WHOQOL-AGE that has been specially adapted for the elderly population. This short version contains 13 of the 100 questions of the original version and has been validated in populations aged over 50 years [25]. Results range from 0 (worst quality of life) to 100 (highest quality of life). Similarly, health-related quality of life was also assessed using the EuroQol 5-D questionnaire (EQ-5D). An adapted version of this questionnaire was used which has been validated in the Spanish population [26]. This questionnaire consists of three elements: the assessment of the individual's health status in levels of severity in different areas (mobility, personal care, daily activities, pain/discomfort and anxiety/depression), the assessment of health status on an analogue visual scale, and finally an index of social values obtained for each state of health generated by the instrument.

##### Other variables

The baseline assessment measured the state of motivation for change described by Prochaska and Diclemente, classified into different stages: Pre-contemplation, Contemplation, Determination, Action, Maintenance and Relapse. App adherence among intervention group participants was measured by the number of records and days with records on the device. A blood sample was taken after a 10–12 h fast to assess laboratory variables including glucose, cholesterol, and glycosylated hemoglobin.

### Data collection procedure, data management and monitoring

The data from the baseline assessment and the 3-month follow-up visits were collected by a specifically trained nurse. The brief counselling after the baseline assessment was provided by another nurse, different from the one collecting the data. Finally, a third member of the research team, not involved in the data collection or brief counselling, was in charge of carrying out the intervention visit. Each participant had a unique identification code within the study. All measurements were compiled in a data collection notebook and kept in a safe place that remained locked within the health centre. A database was created to which only research team members and people related to the statistical analyses had access. The principal investigator or a person designated for this purpose carried out a weekly process of monitoring the study, attending to the inclusion of patients, cleaning and purging of databases, and adaptation of the procedures to the protocol.

### Blinding

Due to the nature of the intervention itself, the participants could not be blinded. However, strategies were implemented to achieve the highest level of blinding possible. The researcher making the visit to the intervention group to explain the app was different from the person charged with making the assessments and the person giving the brief counselling after the baseline assessment visit. Furthermore, the researcher generating the randomization sequence and the researcher in charge of the statistical analysis did not know to which group the participants belonged. To prevent cross-group contamination, no additional advice or reinforcement was given at the 3-month evaluation visit. In addition, the app was not available to the control group for download until the study had finished.

### Statistical analysis

The study data was collected and managed using the REDCap electronic data capture tools, hosted at the University of Salamanca. REDCap (Research Electronic Data Capture) [27] is a secure, web-based software platform designed to support data capture for research studies.

The results are expressed through the mean and standard deviation in quantitative variables or through the distribution of frequencies and percentages in the case of categorical variables. The normality of the variables was evaluated using the Kolmogorov–Smirnov test. In cases where a normal distribution could not be assumed, the corresponding non-parametric tests were used. The chi-square test or Fisher's exact test was used to analyze the association between independent qualitative variables. Means between the two groups were compared using Student's t or Mann Whitney U tests. The relationship between quantitative variables was analyzed using Pearson's correlation coefficient or Spearman's correlation coefficient.

Results of the primary variable and the secondary variables were analyzed by intention to treat (Includes all subjects who are randomized according to the randomized treatment assignment. It ignores noncompliance, protocol deviations, withdrawals, and anything that happens after randomization). ANCOVA was used (model terms time * group interaction) to analyze the effects of the intervention on the primary (number of steps) and secondary outcome, with adjustment for variables showing significant differences between both groups at baseline assessment (age, educational level and health centre). For the three variables measuring cognitive performance (MMSE, Clock test score and categorical fluency), the baseline MMSE score was also included, which presented statistically significant differences between groups at the beginning of the study.

All analyses were performed with SPSS version 23.0 (IBM Corporation, Armonk, NY, USA), with an alpha risk of 0.05 set as the limit of statistical significance.

## Results

### Characteristics of the study population

The study population comprised 160 participants (IG = 81, CG = 79), with a mean age of 70.8 years (SD 4.0), and 61.3% women. Those who were married or living with other people made up 71.9%, with the total number of cohabitants in the house being 2.0 ± 1.0, including the participant. Primary education had been completed by 47.5%, while 19.4% had completed higher education. Regarding risk factors, arterial hypertension and dyslipidemia predominated, being found in 49.4% and 51.2% of the sample, respectively. Treatment for anxiety and depression was being received by 8.8% and 12.1%, respectively. The mean Charlson Comorbidity Index score was 3.1 ± 1.1. With regard to motivational state, assessing the state of change in dietary habits, a total of 138 people (86.3%) were in the maintenance stage, while only four (2.5%) were in the preparation stage. In terms of motivation to change physical activity levels, 122 people (76.3%) were in maintenance and 11 (6.9%) in preparation stages.

There was a difference in the mean age between the IG (69.9 ± 3.6) and the CG (71.7 ± 4.2), *p* = 0.004, and in educational level (*p* = 0.006) (Table 1).

**Table 1:** Baseline characteristics of the study population

	**Intervention group (*n* = 81)**	**Control group (*n* = 79)**	***p* value**
Age (years)*	69.9 (3.6)	71.7 (4.2)	0.004
Females (n, %)	50 (61.7%)	48 (60.8%)	0.900
Marital status (n, %)			0.169
Single	2 (2.5%)	8 (10.1%)	
Married	62 (76.5%)	53 (67.1%)	
Separated or divorced	6 (7.4%)	4 (5.1%)	
Widower	11 (13.6%)	14 (17.7%)	
Number of cohabitants (number)	2.1 (1.1)	2.0 (0.9)	0.264
Educational level completed (n, %)*			0.006
University studies	24 (29.6%)	7 (8.9%)	
Middle or High school	22 (27.2%)	27 (34.2%)	
Elementary school	35 (43.2%)	41 (51.9%)	
None	0 (0.0%)	4 (5.0%)	
Dietary habits stage of change (n, %)			0.676
Precontemplation	3 (3.7%)	1 (1.3%)	
Contemplation	5 (6.2%)	8 (10.0%)	
Preparation	3 (3.7%)	1 (1.3%)	
Action	0 (0.0%)	1 (1.3%)	
Maintenance/termination	70 (86.4%)	68 (86.1%)	
Physical activity stage of change (n, %)			0.293
Precontemplation	7 (8.6%)	7 (8.9%)	
Contemplation	8 (9.9%)	3 (3.8%)	
Preparation	6 (7.4%)	5 (6.3%)	
Action	0 (0.0%)	2 (2.5%)	
Maintenance/termination	60 (74.1%)	62 (78.5%)	
Diabetes (n, %)	16 (19.8%)	19 (24.1%)	0.511
Dyslipidemia (n, %)	43 (53.1%)	39 (49.4%)	0.638
Hypertension(n, %)	34 (42.0%)	45 (57.0%)	0.058
Depression (n, %)	9 (11.1%)	15 (19.0%)	0.162
Anxiety (n, %)	11 (13.6%)	12 (15.2%)	0.822
Charlson Comorbidity Index	2.9 (1.1)	3.2 (1.0)	0.073
Smokers (n, %)	1 (1.2%)	4 (5.1%)	0.164
Obesity (n, %)	20 (24.7%)	27 (34.2%)	0.170
Antiaggregants drugs (n, %)	6 (7.4%)	14 (17.7%)	0.052
Anticoagulants drugs (n, %)	4 (4.9%)	3 (3.8%)	0.737
Hypothyroidism treatment (n, %)	12 (14.8%)	6 (7.6%)	0.148
Lipd-lowering drugs (n, %)	32 (39.5%)	36 (45.6%)	0.438
Antihypertensive drugs (n, %)	34 (42.0%)	42 (53.2%)	0.156
Antidiabetic drugs (n, %)	14 (17.3%)	15 (19.0%)	0.780
Nonsteroidal anti-inflammatory drugs (n, %)	9 (11.1%)	8 (10.1%)	0.884
Anxiety drugs (n, %)	6 (7.4%)	8 (10.1%)	0.543
Depression drugs (n, %)	8 (9.9%)	11 (13.9%)	0.481

Categorical variables are expressed as n (%) and continuous variables as mean ± standard deviation

^*^Statistically significant differences (*p* < 0.05)

### Adherence to the intervention (degree of use of the EVIDENT 3 smartphone application)

The mean number of days the application was used by the IG was 70.7 ± 21.3, an adherence to the intervention of 78.5% ± 23.7%.

### Changes in lifestyles (physical activity and adherence to the MD)

No difference was found in the variables analyzed that could be attributed to either group. (physical activity (steps/min -0.4 (-1.0 to 0.2) *p* = 0.174), and dietary habits (Mediterranean diet score 0.0 (-0.6 to 0.6) *p* = 0.956) that could be attributed to either group after an ANCOVA test (Table 2).

**Table 2:** Physical activity and Mediterranean diet and adjusted intervention attributable difference

Outcome measure	Intervention group (*n* = 81)		Control group (*n* = 79)		Intervention attributable difference (IG-CG) (mean difference (95% CI))	*p* value	F statistic
	Baseline	3 months	Baseline	3 months			
Physical activity- IPAQ-METS/min/week	2473 (1890)	2400 (1960)	2465 (1768)	2283 (1548)	180 (-363 to 724)	0.513	0.430
Hours sitting per week	42.1 (13.1)	45.2 (11.7)	43.9 (10.3)	44.8 (9.8)	0.6 (-3.3 to 4.6)	0.759	0.095
Hours sitting viewing screens per week	37.3 (15.5)	41.7 (13.3)	41.2 (11.1)	40.9 (12.5)	2.6 (-2.1 to 7.2)	0.280	1.175
Average kcal per day	476.3 (310.8)	421.1 (247.9)	414.4 (194.0)	413.4 (213.4)	-35.3 (-106.7 to 36.2)	0.330	0.956
Accelerometer							
% Sedentary	81.8 (4.4)	82.9 (4.5)	81.9 (4.3)	82.6 (5.0)	-0.2 (-1.5 to 1.2)	0.788	0.072
% Light	13.8 (3.2)	13.0 (3.6)	14.0 (4.0)	13.3 (4.1)	0.4 (-0.8 to 1.6)	0.528	0.401
% Moderate	4.4 (2.7)	4.1 (2.8)	4.0 (2.3)	4.0 (2.2)	-0.2 (-0.7 to 0.3)	0.421	0.652
% Vigorous	0.04 (0.08)	0.03 (0.08)	0.05 (0.13)	0.06 (0.19)	-0.01 (-0.07 to 0.05)	0.821	0.051
% Very vigorous	0.00 (0.00)	0.00 (0.00)	0.00 (0.01)	0.00 (0.00)	0.00 (-0.00 to 0.01)	0.248	1.346
% Moderate-Vigorous	4.4 (2.7)	4.1 (2.5)	4.1 (2.3)	4.1 (2.2)	-0.2 (-0.7 to 0.3)	0.409	0.686
Steps per minute	7.4 (3.3)	7.0 (3.4)	6.8 (2.4)	6.6 (2.5)	-0.4 (-1.0 to 0.2)	0.174	1.867
Number of adherence criteria to the MD	8.3 (2.0)	8.5 (1.8)	8.1 (1.7)	8.3 (1.6)	0.0 (-0.6 to 0.6)	0.956	0.003

Intervention attributable difference adjusted for age, educational level and centre

*METS* Metabolic equivalents, *MD* Mediterranean Diet

The numbers of steps/minute, the main objective measure of physical activity, decreased in the IG did so at (-0.6 (-1.0 to -0.2) *p* = 0.003), while the CG did not (-0.2 (-0.6 to 0.2) *p* = 0.194). Further decreases were noted in kcal/day in the IG: (-59.5 (-108.7 to -10.3) *p* = 0.026); while the CG did not (-24.3 (-73.5 to 24.9) *p* = 0.364). The same thing happens in the percentage of time spent in moderate or vigorous activities IG: (-0.4 (-0.7 to -0.0) *p* = 0.017), CG: (-0.2 (-0.5 to 0.2) *p* = 0.480). A non-significant decrease in self-reported physical activity was observed, measured with the IPAQ and analyzed with METS/min/week IG: (-67.5 (-440.8 to 305.8) *p* = 0.647); CG: (-213.3 (-591.7 to 165.1) *p* = 0.213). Regarding sedentary lifestyle results, an increase in the number of hours per week sitting in front of screens stands out was only observed in the IG: (3.5 (0.3 to 6.7) *p* = 0.024; CG: 0.6 (-2.6 to 3.9) *p* = 0.972). Finally, a non-significant increase of 0.2 in the total score of the MD adherence test was observed in both groups.

### Changes in blood pressure, body composition and laboratory variables

No difference was found in the variables analyzed that could be attributed to either group (Table 3). Systolic blood pressure figures decreased in the IG: (-3.7 mmHg (-6.6 to -0.9) *p* = 0.023); while whereas it didn't in the CG: (-1.9 (-4.8 to 1.1) *p* = 0.184), the same occurs in the diastolic blood pressure IG: (-2.2 mmHg (-4.0 to—0.4) *p* = 0.037), CG: (-0.6 (-2.5 to 1.2) *p* = 0.396). Among the anthropometric and body composition parameters, there was a non-significant decrease in both groups in the BMI IG: (-0.3 kg/m2(-0.6 to -0.0) *p* = 0.086); CG: (-0.1 (-0.5 to 0.2) *p* = 0.168. A decrease was observed only in the IG in the body fat percentage IG: (-0.6 (-1.2 to -0.0) *p* = 0.010); CG: (-0.2 (-0.8 to 0.4) *p* = 0.456). Finally, among the laboratory variables related to cardiovascular risk, a decrease in glucose was observed in both groups IG: (-5.5 mg/dL (-9.0 to -1.9) *p* = 0.003); CG: (-5.5 (-9.0 to -2.0) *p* < 0.001) and a non-significant decrease in total cholesterol figures IG: -5.0 mg/dL (-10.6 to 0.7) *p* = 0.088); CG: (-4.9 (-10.6 to 0.7) *p* = 0.071).

**Table 3:** Blood pressure, body composition and laboratory tests and adjusted intervention attributable difference

Outcome measure	Intervention group (*n* = 81)		Control group (*n* = 79)		Intervention attributable difference (IG-CG) (mean difference (95% CI))	*p* value	F statistic
	Baseline	3 months	Baseline	3 months			
Systolic blood pressure (mmHg)	129.6 (17.3)	125.7 (18.5)	134.1 (17.2)	131.6 (17.9)	-2.0 (-6.2 to 2.3)	0.357	0.854
Diastolic blood pressure (mmHg)	78.6 (9.5)	76.4 (9.1)	79.2 (8.6)	78.3 (8.5)	-1.6 (-4.2 to 1.1)	0.243	1.372
BMI (Body mass index) (Kg/m^2^)	28.2 (4.2)	27.8 (4.3)	28.5 (4.4)	28.3 (4.6)	-0.2 (-0.6 to 0.3)	0.447	0.580
Waist circumference (cm)	95.3 (10.7)	94.7 (10.6)	95.6 (10.4)	95.5 (11.0)	-0.5 (-1.9 to 1.0)	0.547	0.364
Hip circumference (cm)	103.8 (9.1)	103.2 (9.1)	104.4 (9.3)	104.2 (10.5)	-0.1 (-1.5 to 1.4)	0.945	0.005
BFM (Body Fat Mass) (Kg)	27.2 (7.3)	26.6 (7.5)	26.9 (8.6)	26.2 (8.7)	-0.6 (-1.3 to 0.1)	0.092	2.883
PBF (Percent Body Fat) (%)	37.3 (7.5)	36.8 (7.9)	37.3 (9.1)	36.4 (9.0)	-0.4 (-1.2 to 0.5)	0.388	0.750
FFM (Fat Free Mass) (Kg)	45.4 (8.3)	45.3 (8.4)	44.4 (7.9)	44.8 (8.2)	-0.2 (-0.7 to 0.4)	0.505	0.446
SMM (Skeletal Muscle Mass) (Kg)	24.7 (5.0)	24.6 (5.0)	24.1 (4.7)	24.4 (4.8)	-0.2 (-0.5 to 0.2)	0.343	0.904
TBW (Total Body Water) (Kg)	33.4 (6.1)	33.3 (6.2)	32.7 (5.8)	33.0 (6.0)	-0.2 (-0.6 to 0.2)	0.374	0.795
Estimated Proteins (Kg)	8.8 (1.7)	8.8 (1.7)	8.6 (1.6)	8.7 (1.6)	-0.1 (-0.2 to 0.0)	0.246	1.355
Estimated Minerals (Kg)	3.2 (0.5)	3.2 (0.5)	3.1 (0.5)	3.1 (0.5)	-0.0 (-0.1 to 0.0)	0.604	0.270
Glucose (mg/dL)	101.1 (22.3)	96.0 (17.1)	104.9 (19.5)	99.6 (18.7)	0.0 (-5.2 to 5.2)	0.991	0.000
Total cholesterol (mg/dL)	194.0 (34.1)	189.6 (38.4)	196.1 (37.6)	188.0 (34.5)	-0.1 (-8.5 to 8.2)	0.976	0.001
HDL-Cholesterol (mg/dL)	57.1 (13.3)	56.7 (13.3)	58.9 (12.8)	58.4 (13.3)	0.8 (-2.1 to 3.8)	0.581	0.307
LDL-Cholesterol (mg/dL)	114.8 (29.8)	112.9 (32.0)	116.8 (35.4)	109.0 (30.1)	1.7 (-6.2 to 9.6)	0.665	0.188
Triglycerides (mg/dL)	115.9 (52.7)	110.0 (42.3)	119.7 (64.4)	119.5 (62.9)	-12.8 (-26.7 to 1.1)	0.071	3.311
HOMA-IR	2.84 (1.64)	2.23 (1.30)	2.85 (2.05)	2.39 (1.87)	-0.19 (-0.73 to 0.35)	0.486	0.489
Glycosylated hemoglobin (%)	5.84 (0.73)	5.91 (1.36)	5.88 (0.71)	5.86 (0.61)	0.1 (-0.3 to 0.5)	0.534	0.389

Intervention attributable difference adjusted for age, educational level and centre

*HOMA-IR* Homeostasis assessment model for insulin resistance

### Changes in cognitive performance, quality of life and instrumental activities of daily living

The groups were comparable in terms of baseline assessment in all variables analyzed with the exception of the baseline MMSE score. In an analysis adjusted for age, educational level and baseline MMSE score, a difference attributable to the intervention was observed in the total Clock test score (0.7 (0.1 to 1.2) *p* = 0.018), while neither the MMSE score nor differences between groups are observed in categorical fluency (Table 4).

**Table 4:** Cognitive performance, activities of daily living and quality of life and adjusted intervention attributable difference

Outcome measure	Intervention group (*n* = 81)		Control group (*n* = 79)		Intervention attributable difference (IG-CG) (mean difference (95% CI))	*p* value	F statistic
	Baseline	3 months	Baseline	3 months			
MMSE score*^,b^	28.7 (1.4)	28.9 (1.1)	27.6 (2.0)	27.9 (2.0)	0.4 (-0.1 to 0.9)	0.108	2.619
Categorical fluency (words/min)^b^	16.8 (4.5)	17.7 (4.9)	16.6 (4.4)	20.7 (4.6)	-4.8 (-10.8 to 1.2)	0.116	2.507
Total score Clock test^b^	9.0 (1.4)	9.3 (1.1)	8.8 (1.4)	8.7 (1.7)	0.7 (0.1 to 1.2)	0.018	5.744
Total score Functional Activities Questionnaire^a^	0.11 (0.42)	0.04 (0.20)	0.12 (0.33)	0.03 (0.23)	0.01 (-0.11 to 0.13)	0.874	0.025
Total score WHOQOL Age test^a^	55.0 (6.4)	55.0 (6.9)	53.4 (7.1)	54.2 (7.3)	-0.9 (-.2.6 to 0.8)	0.285	1.150
EQ-5D-3L score^a^	0.790 (0.189)	0.809 (0.211)	0.783 (0.209)	0.799 (0.185)	-0.001 (-0.053 to 0.051)	0.959	0.003
EQ-VAS (EuroQoL visual analogue scale)^a^	75.8 (15.5)	75.4 (16.3)	72.0 (18.0)	74.5 (17.3)	-1.5 (-6.6 to 3.5)	0.551	0.357

*MMSE* Mini-Mental State Examination

^*^p < 0.05 at baseline evaluation between groups

^a^Intervention attributable difference adjusted for age, educational level and centre

^b^Intervention attributable difference adjusted for age, educational level, centre and MMSE score at baseline

No difference attributable to either group was found in the quality of life and instrumental activities of daily living variables.

## Discussion

In a sample of people aged over 65 years, the combined use over 3 months of the EVIDENT 3 smartphone application and an activity tracking bracelet (smartband), compared to brief lifestyle advice, did not change lifestyles in terms of the amount and level of physical activity or eating habits. Nor were changes observed in other clinical parameters such as blood pressure, body composition and laboratory variables related to cardiovascular risk. At the cognitive level, a slight improvement was seen in the Clock test score, but without changes in the MMSE score or in the categorical fluency test. Lastly, neither quality of life nor independence in carrying out activities of daily living were affected by the intervention performed.

The EVIDENT study is a series of combined studies evaluating the effectiveness of using the EVIDENT Smartphone application in modifying lifestyles in different subpopulations. Although physical activity of moderate or vigorous intensity increased more in the application + counselling group than in the counselling-only group, the results of the EVIDENT 2 study, conducted in the general population, found no differences when comparing the increase between the two groups [28]. Likewise, although with some change in body composition variables, the EVIDENT 3 study, conducted in an overweight or obese population, did not show relevant changes in the amount and intensity of physical activity or in nutritional habits in the group that used the app and the smartband compared to the control group [29]. Only a population of older people with type 2 diabetes mellitus obtained beneficial results in terms of increased physical activity and better adaptation to the MD [11, 30]. The results of the EVIDENT-AGE study similarly fail to show that the combined use of the EVIDENT 3 smartphone application and a physical activity tracking bracelet produced changes in physical activity levels and intensity or in the eating habits of a sample of people aged over 65 years. Nevertheless, the results shown in this study prompt deep reflection on some issues. First of all, the characteristics of the smartphone application itself. The EVIDENT smartphone app is analytical, in the sense that it generates a set of recommendations based on the user’s information input with the aim of enabling the user to modify those lifestyles aspects not meeting the minimum requirements. However, the development of this app did not take other motivational frameworks into account such as the social framework or the affective framework. The study by King A. et al. [31] assessed the effect of three apps with different characteristics for sedentary lifestyle over eight weeks. The app that was based on a social framework and encouraged social interaction was the one that obtained the best results in terms of increasing physical activity and reducing sedentary lifestyle. The success of interventions using m-Health in older adults depends, to a large extent, on the motivational state and the support they receive for its use [8]. In the EVIDENT-AGE study, more than 75% of the participants were in the maintenance stage of change for modifying physical activity or eating habits, while very few (3, 7%) were in the preparation stage, according to the stages of change described by Prochaska et al. [32, 33]. This aspect could therefore have been decisive in the results found since many participants were perhaps already meeting the requirements to a reasonable degree. Another important aspect to take into account is that the EVIDENT 3 smartphone app does not have an interface specifically designed for older people, which may have resulted in difficulties in the learning process. In older people, the most difficult barrier to overcome regarding the use of technology is the prior ignorance of how such applications work, which can be overcome by working as a team with the community as a whole, especially in the health and education sectors, as well as in the family or social nucleus [34].

No relevant changes were observed in clinical parameters of body composition or lifestyle-related laboratory variables, although a non-significant difference was observed which could mark a trend towards a greater decrease in blood pressure figures in the IG. A recent systematic review on this topic [5] concluded that the evidence on the effectiveness of e-Health or m-Health interventions in healthy aging is still limited, although some studies show beneficial results, especially in some clinical parameters such as blood pressure.

The use of memory training programs through apps designed for mobile phones has, in some cases, achieved clinically relevant increases in older people of working memory and attention, which are among the most important cognitive capacities [35]. In the EVIDENT-AGE study, a modest but promising increase in the total score on the Clock test was achieved. This test assesses a variety of cognitive functions, such as understanding of verbal command, memory, spatial coding knowledge, as well as fostering constructive skills. However, no results favourable to the intervention were found on the MMSE, although this is a screening tool for dementia and not an appropriate outcome tool. Lastly, significant changes were not observed in health-related quality of life or in independence in carrying out instrumental activities of daily living, probably because the level of independence of the selected sample was already very high at baseline (Total score Functional Activities Questionnaire 0.11 ± 0.38). A further limitation is related to the level of motivation, since most of the participants were in the maintenance stage, which makes it impossible to modify their habits, so the possibility of applying this intervention in subjects with less healthy habits is suggested.

In addition to those mentioned above, a limitation of a methodological nature should also be noted. The very nature of the intervention makes it impossible to blind the participants, although we have tried to reduce the impact of this situation through certain measures, such as blinding the researcher carrying out the assessments. Another possible limitation in the interpretation of the results would be the difference found in the baseline measurement in the age of the participants and in the educational level that could condition the results, although it has been partially addressed by including these variables in the adjustment models. Finally, note that the sample was not a random sample of individuals from the community, so the results should be taken with caution because they may not represent the community of people over 65 years of age.

Since the level of adherence to the application was generally high, and since no positive results were found in the variables analyzed, it would be necessary to know, through qualitative research, what were the difficulties encountered in the use of the smartphone application. and in its maintenance in the medium and long term and the possible reasons that may have occurred so that its use does not increase physical activity.

## Conclusions

In a sample of people over 65 years of age, the combined use of the EVIDENT 3 smartphone app and an activity tracking bracelet (smartband) for 3 months did not result in lifestyles changes related to the amount and level of physical activity or the eating habits, compared to brief lifestyle advice. Other clinical parameters were not changed either, although at the cognitive level, a slight improvement was observed in the score on the Clock test assessing a variety of cognitive functions such as memory.

## Data Availability

The datasets used and/or analysed during the current study are available from the corresponding author on reasonable request.
